# Model depicting aspects of audit and feedback that impact physicians’ acceptance of clinical performance feedback

**DOI:** 10.1186/s12913-016-1486-3

**Published:** 2016-07-13

**Authors:** Velma L. Payne, Sylvia J. Hysong

**Affiliations:** Houston Center for Innovations in Quality, Effectiveness & Safety, Michael E. DeBakey Veterans Affairs Medical Center, 2002 Holcombe Blvd (MEDVAMC 152), Houston, TX 77030 USA; Baylor College of Medicine, Houston, TX USA

**Keywords:** Audit and feedback, Feedback acceptance, Performance improvement

## Abstract

**Background:**

Audit and feedback (A&F) is a strategy that has been used in various disciplines for performance and quality improvement. There is limited research regarding medical professionals’ acceptance of clinical-performance feedback and whether feedback impacts clinical practice. The objectives of our research were to (1) investigate aspects of A&F that impact physicians’ acceptance of performance feedback; (2) determine actions physicians take when receiving feedback; and (3) determine if feedback impacts physicians’ patient-management behavior.

**Methods:**

In this qualitative study, we employed grounded theory methods to perform a secondary analysis of semi-structured interviews with 12 VA primary care physicians. We analyzed a subset of interview questions from the primary study, which aimed to determine how providers of high, low and moderately performing VA medical centers use performance feedback to maintain and improve quality of care, and determine perceived utility of performance feedback.

**Results:**

Based on the themes emergent from our analysis and their observed relationships, we developed a model depicting aspects of the A&F process that impact feedback acceptance and physicians’ patient-management behavior. The model is comprised of three core components – Reaction, Action and Impact – and depicts elements associated with feedback recipients’ reaction to feedback, action taken when feedback is received, and physicians modifying their patient-management behavior. Feedback characteristics, the environment, external locus-of-control components, core values, emotion and the assessment process induce or deter reaction, action and impact.

Feedback characteristics (content and timeliness), and the procedural justice of the assessment process (unjust penalties) impact feedback acceptance. External locus-of-control elements (financial incentives, competition), the environment (patient volume, time constraints) and emotion impact patient-management behavior. Receiving feedback generated intense emotion within physicians. The underlying source of the emotion was the assessment process, not the feedback. The emotional response impacted acceptance, impelled action or inaction, and impacted patient-management behavior. Emotion intensity was associated with type of action taken (defensive, proactive, retroactive).

**Conclusions:**

Feedback acceptance and impact have as much to do with the performance assessment process as it does the feedback. In order to enhance feedback acceptance and the impact of feedback, developers of clinical performance systems and feedback interventions should consider multiple design elements.

## Background

Audit and feedback (A&F) is a strategy that has been used across various disciplines for performance and quality improvement [1–9]. Extensive research exists linking feedback characteristics to performance improvement [2, 3, 5–7, 9]. Additionally, research has shown that feedback has varying impact on patient outcomes and changing health professionals’ behavior [5]. Limited research exists regarding medical professionals’ uptake or response to clinical-performance feedback and whether feedback impacts how clinicians manage patients, two important contextual components of feedback [7, 10]. With less-than-optimal clinical outcomes in some cases even after receiving feedback, [5] there is a need to investigate the mind of the feedback recipient to determine factors associated with acceptance and alteration of patient-management behavior to enhance performance. An understanding of these factors will enable development of strategies to provide clinicians with actionable, impactful feedback.

The objectives of this research are to determine if there are aspects of the audit and feedback process that impact physicians’ acceptance of clinical performance feedback. We also sought to determine actions physicians take when receiving performance feedback, and if receiving feedback results in physicians altering their patient-management behavior.

## Methods

### Study design

The Baylor College of Medicine Institutional Review Board approved this study (H-20386). We performed a secondary qualitative analysis of interviews with 12 primary care physicians (PCPs) practicing in geographically dispersed Veterans Affairs Medical Centers (VAMCs). We analyzed all of the physician interviews collected during the primary study. The objectives of the primary study were to compare how leaders and clinicians of high, low and moderately performing VAMCs use clinical performance data from the Veterans Affairs External Peer Review Program (EPRP) as a feedback tool to maintain and improve quality of care. Reference Morgan, et al. for further details on the VAMC system [11]. The present study focused on phenomena affecting the acceptance of said feedback by physicians specifically, as well as their subsequent behavioral reactions.

### Participants

Interview transcripts from 12 full-time PCPs who practiced at least 3 years in their current position; new or part-time physicians were not included due to limited exposure to the clinical-performance and feedback process. During the primary study, physicians from each site were randomly selected from those meeting eligibility criteria. Since the primary study was a site comparison analysis, demographic and characteristic data of individual interviewees was not collected. Site characteristics are included in Appendix A; site-selection and data-collection methods are described elsewhere [4]. Participants were emailed a document analogous to a consent form to review prior to the interview. Verbal consent was obtained and audio-recorded at the beginning of the interview; consent recordings were stored separate from the interview audio recording.

### Data collection

During the primary study, participants answered questions about (a) type of EPRP information received, (b) type of quality/clinical performance information physicians seek, (c) opinions and attitudes regarding EPRP utility, (d) how EPRP data is used, and (e) sources of information or strategies used to improve performance. EPRP is a nationally abstracted database containing performance data for all VA medical facilities on over 90 indicators including access, quality of care, cost effectiveness, and patient-satisfaction; data are abstracted monthly and reported quarterly. The interview guide used in the primary study is included in Appendix B. For the present study, we focused on a subset of questions that best addressed the research questions of interest (see data analysis section below). The interviews from the primary research study provided data depicting physicians’ perspectives of the value of the VA clinical performance process. These interviews also enabled us to investigate if there are aspects of this process that impact feedback acceptance and change their patient-management behavior.

### Data analysis

We utilized techniques from grounded-theory and content-analysis [12, 13] methodologies to analyze physician responses to three of the 16 questions collected during the primary study. Coding was facilitated using Atlas.ti qualitative data analysis software.

To obtain an overall understanding of the data, transcripts were read in their entirety by both authors. We determined responses to the following questions best addressed our research objectives.

1. In your efforts to provide the highest quality of care, how do you go about assessing the quality of care you provide?
2. What do you do with feedback you receive?
3. What can your facility do, that they are not doing, to help you track your performance?

The first author (VLP) coded transcripts deductively looking for statements related to physicians’ perception of the value of feedback, feedback acceptance, action taken on feedback, and the impact of feedback on patient-management behavior. We defined *feedback acceptance* as acknowledgment that feedback offers insight on improvement areas that may enhance clinical performance and/or patient outcomes. *Non-acceptance* was defined as the belief that feedback has no positive impact on clinical performance and/or patient outcomes. Upon completion of coding ten percent of the transcripts, a codebook was developed, which was referred to throughout the coding process. Transcripts were analyzed line-by-line using an open coding and constant comparative approach; the codebook was refined as necessary. We reached thematic saturation after eight interviews; however, given the small number of available interviews, we included data from all interviews for completeness. During the axial coding process performed by both authors, we categorized similar codes, thematically organized them and discovered relationships between emerging themes. Once we determined the ‘what’ associated with feedback acceptance, to begin forming our model we started to investigate ‘why’ by exploring depth, context and variation (dimension). These were explored by looking at theme groundedness and variation of the concepts across and between participants [12, 14, 15]. We reviewed the frequency of codes and performed a multi-step process similar to the context, paradigm and conditional/consequential matrix processes described by Corbin and Strauss [12]. We investigated the concepts expressed by the majority of participants and sought the answer ‘why’, ‘where’, ‘how’, and ‘what happens’; we investigated actions, interactions, emotion and consequences. We identified and looked for key words (e.g., because, when, we do/don’t, it makes you feel) that provided clues to actions, explanations and emotion, and followed these through the data to determine antecedents and consequents. To explore variation we investigated themes expressed by only a few of the participants using the same techniques. The interviews contained a considerable amount of emotion, which led us to investigate physicians’ adamancy regarding certain topics. We reviewed the number of times physicians returned to a particular topic during the interview and determined the number of physicians who discussed the topic. Throughout these processes, we explored high-level (macro) concepts and how lower-level (micro) explanatory concepts to explain relationships between individuals, physician groups and VA facilities [12]. During this selective coding process we found that there was a theoretical structure associated with the core phenomenon of feedback acceptance. It was clear there were definite aspects of the A&F process that impacted feedback acceptance, and there were antecedents and consequents of acceptance. This led to the development of a model regarding physician feedback acceptance based on the themes and their interrelationships that emerged from the data. This data analysis strategy is consistent with processes specified by qualitative data analysis experts including Corbin and Strauss [12], Miles and Huberman [15]. Hysong and colleagues also utilized this approach when creating an emergent model based on secondary analysis of interview data [3].

## Results

**Fig. 1:**
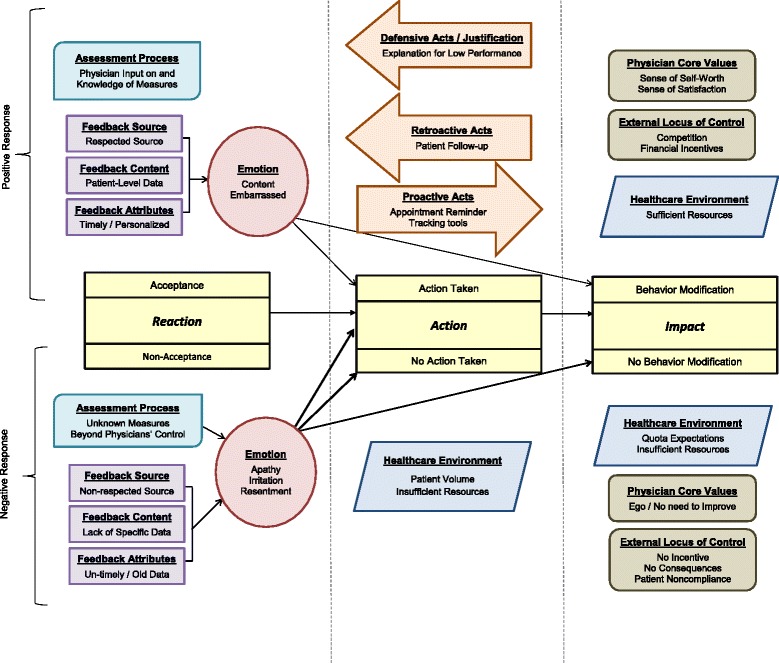
Model Depicting Impact of Performance Feedback on Physician Patient-Management Behavior. Color and shapes are used to convey factors that are alike and different. Arrows depict relationships between factors. Line thickness depicts strength of the relationship of the components

During our analysis several themes emerged associated with facets of the A&F process that, for this group of physicians, impacted their acceptance of and response to feedback, which in turn impacted their patient-management behavior. This formed the foundation for our model of physician feedback acceptance, detailed below and graphically depicted in Fig. 1.

### Physician feedback model

The physician feedback model has three components - Reaction, Action and Impact. *Reaction* relates to physicians’ acceptance of clinical-performance feedback. *Action* relates to behaviors physicians actually engage in after receiving and reacting to feedback. *Impact* relates to the effect feedback has on patient-management behavior. Aspects of A&F that induce or deter reaction, action and impact (depicted above and below the components listed in the center row of Fig. 1) include feedback features, the assessment process, environmental factors, core values, external locus of control and emotion. Arrows illustrate relationships and line thickness represents strength of the relationship between components. Thicker lines indicate a stronger relationship determined by theme groundedness in the data. That is, thicker lines depict a greater number of codes and quotations associated with a theme than themes depicted by thinner lines. Table 1 contains a matrix of aspects of A&F related to each component. Table 2 provides an overview of the aspects of A&F that induce and deter feedback acceptance, along with the groundedness of the emerging themes.

**Table 1:** Feedback acceptance component / aspect of audit and feedback matrix

Aspect of A&F	Component 1 Reaction	Component 2 Action	Component 3 Impact
Feedback Features	X		
Assessment Process	X	X	X
Emotion	X	X	X
Environment		X	X
External Locus of Control			X
Core Values			X

**Table 2:** Aspects of A&F affecting feedback acceptance, action and impact

Topic	Number of Providers that Discussed Topic (Theme Groundedness) N (%)	N Times Topic Discussed in a Single Interview † (Min - Max)
External Locus-of-Control		
Financial Incentives (+)	11 (92)	1–3
Non-Compliant Patients (-)	8 (67)	1–5
Competition (+)	6 (50)	1–5
Lack of Consequences (-)	6 (50)	1–3
No Recognition of “Job Well Done” (-)	4 (33)	1–7
Management Style / Style of Delivery (+,-)	3 (25)	1–16
Emotion		
Frustration / Irritation (-)	8 (67)	1–9
Apathy (-)	6 (50)	2–5
Resentment (-)	4 (33)	1–3
Contentment / Pride (+,-)	4 (33)	1–5
Discouragement / Humility (+,-)	3 (25)	1–2
Embarrassment / Shame (+)	2 (17)	1–2
Procedural Justice of Assessment Process		
Unfairly Penalized (-)	10 (83)	1–14
Small Sample Charts Reviewed (-)	6 (50)	1–6
Unaware of Measures Being Tracked (-)	4 (33)	1–6
Feedback Features		
Feedback Content (+,-) Aggregated vs. Personalized Data	8 (67)	1–16
Feedback Temporality (+,-) Timely vs. Untimely Delivery	7 (58)	1–13
Feedback Source (+,-) Respected vs. Not Respected Individual	3 (25)	1–6
Environment		
Time Constraints / Patient Volume (-)	10 (83)	2–8
Inadequate Resources (-)	4 (33)	1–4
Quality Clinical Team (+)	1 (17)	1–2
Stress / Cognitive Overload / Burn-out (-)	2 (25)	1–3
Core Values		
Desire to Help Patients (+)	8 (67)	1–4
Performance Good Enough (-)	4 (33)	1–4

+ Positive Impact

- Negative Impact

† Counts include the initial mention of the topic and each subsequent mention (return to) the topic after discussion of a different topic

### Component 1 – Reaction

Aspects of the A&F process that impact recipients’ initial reaction to feedback are associated with characteristics of the feedback, the assessment process and emotion.

#### Feedback characteristics

Feedback characteristics that impact acceptance include *content*, *temporality* and *source*. Feedback that provides individual physician data identifying specific improvement areas based on recent patient visits, delivered by an individual that is familiar with the clinical environment, was more favorably received than aggregate-level data based on old data delivered by someone unfamiliar with what physicians deal with day-to-day:

*We complained a few months ago that we wanted to see more individualized provider reports … There’s no provider-specific data. (Physician 9).*

*They screen performance so frequently you don’t stay in a downward trend for long. They identify which plates are about to fall, and they’ll let you know. I’d rather they do that than one day come in and say, ‘Hey, your performance measures over the past 6 months totally suck’ (Physician 6).*

*They don’t understand how many hours we put in here. They don’t understand how many patients we have … They don’t understand the work involved with each patient. They just care that your numbers are down. (Physician 8).*

#### Performance assessment process

The performance assessment process can negatively impact acceptance especially when physicians feel their performance is based on a small sample of patients that are not representative of the care they provide, and when they are penalized for factors beyond their control such as noncompliant patients.

*They review Hemoglobin A1C… something that is supposed to be checked yearly. If the patient was not here in the last year or does not go to the lab, how can we check their A1C? We give them the card … you can see in the record it was ordered several times … but they don’t go to the lab. We have no control over patients … they don’t comply… but we get penalized for it. (Physician 9)*

#### Emotion

Feedback can stir an emotional response that impacts acceptance. Physicians feel a sense of pride when receiving feedback that indicates their performance is above average, or embarrassed when low-performing feedback is received. Physicians can become discouraged when management focuses only on suboptimal performance and do not acknowledge a job well done.

*When we receive the monthly report and see how you are doing compared to the other teams, you feel good knowing that you’ve done a good job. (Physician 8)*

Emotional responses can be of varying intensity. Physicians can become irritated, resentful and apathetic when they feel they are penalized for factors beyond their control or when feedback is delivered in a derogatory manner.

#### Component 2 - Action

The second component of the physician feedback model is *Action*. This component refers to the action physicians take when receiving feedback. Themes that emerged regarding action include emotion prompted by feedback and the assessment process, and aspects of the clinical environment that induce or deter the ability to take action. Physician actions take fell into three categories: *Retroactive* (revisiting previously seen patients to correct a problem); *Proactive* (focusing on future patients); or *Defensive* acts (justifying performance) (Table 3). When physicians accept feedback, they commonly take proactive or retroactive acts; whereas not accepting feedback normally results in no action or defensive acts.

**Table 3:** Physician action types

Action Type	Definition	Examples of Actions Taken
Retroactive Acts	The physician revisited previously seen patients to correct an identified problem	Contact patients who did not come in for a scheduled office visit to reschedule the appointment. Contact patients who were given an order for a test or procedure, but has not gotten the test/procedure, to remind them of the importance of the test.
Proactive Acts	The physician focused on future, rather than previously cared for patients	Contact patients who have an upcoming appointment to remind them of the appointment. Physicians developing tools to track their patients and performance. Providing patients with education and/or tools to better manage their disease (i.e. provide them with blood pressure equipment).
Defensive Acts	The physicians attempted to justify their specified level of performance. The defensive approach was often used when physicians felt there was an external locus of control (or factors outside their control such as non-compliant patients) that impacted their performance.	Provide management with an explanation as to why performance was low. This approach often required extensive research to identify and document the factors associated with low performance. Along with the explanation for low performance, many (4 of 12) physicians offered improvement suggestions.

#### Performance assessment process

As with the reaction component, physicians’ disagreement with the assessment process results in no action. When they feel performance is based on a small sample of patients that is not representative of the care they provide they ignore the feedback and do not take any action. In addition, physicians cannot carry out tasks to meet performance expectations when they were not aware of what measures are being tracked.

*The N is incredibly tiny. These patients may not be representative of our typical patient, yet these numbers are taken very seriously. There’s a lot of things on EPRP that don’t always get reported, so we don’t always know what’s being tracked. The major focus has always been on diabetics measures. The other things were not as frequently brought to our attention. (Physician 12)*

#### Emotion

Emotion can be a precursor to action, and emotion intensity can determine type of action. Physicians’ displeasure with the assessment process can result in negative emotion, such as irritation or frustration, which results in inaction. Not accepting feedback and negative emotion are commonly associated with defensive actions. When physicians receive feedback of suboptimal performance or feedback they do not agree with, they tend to react defensively and provide justification for their performance.

*When you receive the list and see you have fallen out, the first step is look at the cases and see if there’s any reason why we should provide a rebuttal. We feel like we have to defend why measures fall out. (Physician 11)*

Negative emotion such as disappointment or humility over low performance can drive action that improves performance.

*[In meetings where performance is openly discussed] You have to be willing to be humble enough to say, ‘Hey, it seems like me and one other person here are the only ones in red all the time, so I would like to know what you all are doin’ to keep levels up?’ … It’s interesting because you get a feel for what others are doin’, so you can correct what you are doin’. (Physician 1)*

Feedback can also trigger positive emotion that induces action. When receiving favorable feedback, physicians feel a sense of pride; and take steps to ensure performance is maintained.

*I think, basically, EPRP helps a lot in defining the criteria. You know where you are standing in relation to others. I think the higher you score, the better you feel. You want to maintain that level so you try to do all you can to keep on top. (Physician 5)*

Positive emotion may also induce inaction if physicians feel there is no need to improve their performance.

*For the most part, my numbers are usually in the middle, around the average… near the bar… I always feel like if I’m within one standard deviation of the bar, I can live with that. (Physician 3)*

#### Environmental factors

Aspects of the clinical environment such as time constraints and patient volume and insufficient resources impair the ability to address feedback.

*The biggest glaring deficit that we have is staffing … We are working with less than a skeleton crew - we’re a skeleton crew minus one rib per skeleton …We are always going to be getting more patients and, if they continue to hold fast to this idea that when one leaves we cannot replace them, we’re gonna be facing a lot of difficulties keeping up with our measurements. I don’t think it’s reasonable. (Physician 6)*

#### Component 3 - Impact

The third component of the physician feedback acceptance model is *Impact,* which is associated with aspects of A&F that have an effect on physicians’ patient-management behavior or incorporating changes into clinical practice to enhance patient care. Several themes emerged that were associated with behavior modification; these included external locus-of-control, core values, emotion and environmental factors.

#### External locus-of-control

Locus-of-control refers to the extent to which an individual believes they can control events that affect them [16]. An individual’s “locus” is conceptualized as internal or external. Individuals with a high internal locus of control believe events in their life derive primarily from their own actions. Whereas, people with an external locus of control praise or blame an external factor for the event; what happens to them is attributable to external forces beyond their influence.

Financial incentives based on performance and competition amongst peers, physician groups and facilities induce behavior modification and often lead to incorporating changes into the clinical setting. The absence of these items resulted in no changes.

*You are in at the end of the day because the performance pay is based on how you perform on your performance measures. (Physician 7)*

*Healthy competition is good. My team does really well with the performance measures. When we get that monthly review and you see how the other teams are doing, you feel good just knowing that you’ve done a good job. It makes you feel better, and you want to do more. (Physician 8)*

The lack of consequences for low performance and no verbal or financial recognition for good performance results in no effort to enhance performance. Physicians, who feel there is nothing they can do to induce patients to comply with their recommendations, do not change their patient-management behavior. They feel any effort made to enhance patient care will be reversed by noncompliant patients, so why bother trying.

The manner in which feedback is delivered is also linked to patient-management behavior modification. When an individual that understands the environment delivers suboptimal performance feedback in an empathetic manner, physicians are more willing to take steps to enhance performance. When feedback was delivered in a derogatory manner, or delivered by an individual that did not understand the environment, physicians are less interested in enhancing performance.

*Sometimes it’s demeaning the way they treat you … you’re an adult after all. That was the way the person that we had before was. It doesn't make you feel very good. So, basically, my mechanism is ignore negative feedback or any sly remarks. I had a situation where there was something I didn’t do. They went to the top boss, and he got angry. Well, the new person is like, “Hey, you know, I wanted to remind you you’ve got to do this. Somehow it went to upper management, and I know it’s kind of embarrassing, but, you know, can you do it?” I’m like, “Yeah, sure”, and I did it that night. It was the same problem, but it was presented in a ‘helping each other out’ manner. (Physician 4)*

#### Core values

Physicians who are driven by a sense of helping patients improve their health and provide quality care strive to make changes when suboptimal performance is reported.

*The reason I provide good care is for the patients, not because I’m gonna get more money. I guess I’m a kind of a purist like that. I wanna provide good care because that’s what we should be doing. (Physician 8)*

#### Emotion

Emotion can lead to patient-management behavior modification. The intensity of the emotion is also a factor associated with behavior modification. Discouragement or embarrassment can help physicians realize they can do better. Negative emotion is often coupled with non-acceptance and results in no behavior modification. When physicians are irritated, frustrated or become apathetic over the assessment process change is less likely.

#### Environmental factors

Even if enhancing performance is important to physicians, they may be unable to incorporate changes due to time constraints, patient volume and quotas, and not having adequate staff to impact change. Conversely, the presence of a well-staffed and quality team is essential to effect change. Stress, information overload and burnout are also factors that impact the ability to incorporate change.

*Primary care is very dynamic and challenging because there’s precious few providers, even fewer coming down the pike, and more and more demands from the patient population burgeoning. We’re getting 500 new patients a month; and the average provider here is running 105-115 % of their expected empanelment, so nobody’s happy because everybody’s being squeezed, and there’s a hiring freeze and the budget is terrible and so on and so forth… (Physician 12)*

## Discussion

From our analysis of physician interviews originally designed to obtain their views on the value of clinical performance feedback, a set of interrelated themes regarding aspects of A&F that impact feedback acceptance emerged. From these themes we developed a model depicting antecedents and consequents of physicians’ acceptance of clinical performance feedback. The model we developed consists of three core components: reaction, action and impact and depicts elements that induce or deter each component. These elements are associated with aspects of the audit process; content and attributes of feedback; aspects of the healthcare environment; physicians’ internal core values; and external locus-of-control.

Based on physicians’ mixed reaction to clinical-performance feedback, physicians are accepting of feedback that is timely, personalized and includes patient-level data that permits them to identify specific areas of improvement. The assessment process can stir resentment and negative emotion that leads to non-acceptance. Physicians ignore feedback when they feel performance is based on small, unrepresentative patient samples, unrealistic measures, and become irritated when they are penalized for factors beyond their control. Feedback acceptance is indicative of action, inaction and type of action. Non-acceptance, coupled with intense negative emotion (i.e., anger, resentment) results in defensive actions; whereas less intense emotion (i.e., embarrassment, humility) result in proactive or retroactive acts. Feedback acceptance and external locus-of-control elements such as financial incentives can lead to patient-management behavior modification. Factors deterring patient-management changes include feedback based on old data, unrealistic measures, and environmental factors such as patient volume, time restrictions and insufficient resources.

Since the release of Kluger and DeNisi’s seminal article indicating that feedback interventions range from positive to debilitating effects on performance, [7] many researchers have tried to identify specific aspects of feedback that cause such outcomes [2–7, 9, 10]. Researchers agree that before feedback can be useful it must be accepted and internalized [17, 18]. This has led to empirical studies on feedback acceptance; however these studies have focused on multisource or 360° feedback, [19, 20] feedback in non-medical domains, [20, 21] and feedback given to clinicians-in-training [22, 23]. Our findings are consistent with and extend previous research, suggesting that feedback characteristics such as content, temporality and source can enhance feedback’s effectiveness [2, 3, 5–7, 10, 24–27]. For example, in a study comparing clinical practice guideline implementation patterns of high- and low-performing VA facilities Hysong, et al. found providers at high-performing facilities received timely, individualized, non-punitive feedback; compared with feedback delivered to providers at low-performing facilities with varying timeliness, consisting of aggregated data [3]. Hysong’s study uncovered a hierarchy of feedback cues associated with impactful feedback, with timeliness at the top, followed by individualization, non-punitiveness and customizability [3]. Similarly, a meta-analysis examining whether FIT-based [7] feedback characteristics explained observed variability in effectiveness of health-care feedback interventions showed that A&F has modest, though significant, positive effect on quality outcomes when feedback is written, frequent and includes specific suggestions for improvement [2]. A systematic review of empirical studies involving baseline and follow-up performance measurement after receiving feedback to determine the impact of feedback on clinical performance revealed feedback can change physicians’ clinical performance if it is provided systematically over an extended period of time (years) by an authoritative, credible source [9]. Several other studies have demonstrated a credible source is imperative to feedback acceptance [24–26]. Our finding of physicians’ concerns regarding the limitations of performance assessment including inaccurate interpretation of performance determined when assessing a small sample of patients, and physicians’ need to validate feedback data are consistent with the findings of Yi, et al. [28]. Aspects of our model are also consistent with longstanding psychological research on locus-of-control [16, 29–34] in that we found external locus-of-control factors such as financial incentives and competition are primary factors inducing physicians’ behavior modification to enhance performance. Whereas, aspects of the clinical environment (i.e., patient volume and time constraints) deter patient-management behavior modification. Our study extends this prior research by establishing the relational link between feedback acceptance and locus-of-control. Our findings further extend prior research in that we sought to penetrate the mind of the feedback recipient, specifically advanced medical professionals, to understand what influences feedback acceptance and induce them to modify their patient-management behavior to enhance performance.

A phenomena of interest associated with feedback acceptance and impact that emerged in our research, not reported in prior research, is that receiving feedback can generate a great deal of emotion in physicians. There are specific emotions that steer physicians toward or away from taking action and modifying their patient-management behavior. Not only does the presence of emotion impact acceptance and behavior modification, the intensity of emotion also plays a role. Understanding what occurs ***within*** feedback recipients when feedback is received can be a critical step toward enhancing the A&F process.

Perhaps most interesting is that the underlying source of physicians’ emotion is not the feedback, but rather the antecedent event. Physicians’ perceived procedural justice of the assessment process (their perceptions of whether or not the assessment process was fair) is what stirred intense emotion leading to not accepting and acting upon the feedback. Being penalized for factors outside their control (i.e., noncompliant patients) and performance being based on a small sample of patients not representative of the care physicians provide, stirs negative emotion that may reverse, or at least minimize, the effect of feedback. Our research reveals that feedback acceptance may have as much to do with knowledge and approval of the assessment process as it does the feedback.

Practical application of our model should start with the realization that feedback can be a self-assessment tool that can help the recipient identify specific areas of improvement [35, 36]. However, when the recipient experiences intense emotion when receiving feedback, it may reduce its potential effectiveness. Preceding feedback delivery with knowledge of the assessment process may heighten feedback acceptance and effectiveness. Involving physicians in the development of performance measures may enhance its effectiveness. Our research provides a model detailing multiple design elements that should be considered when developing feedback interventions designed to enhance performance. Our model not only depicts aspects of the A&F process that impact acceptance, it also provides insight on emotions, core-values and external forces that induce or deter performance.

### Limitations

The main limitation of our study is the use of secondary data not specifically designed for our research objectives. Because we used data from existing interviews, we were limited by the content of the questions asked and the sampling strategy used in the primary study (in this case a purposive sampling strategy for maximum variation among sites and among personnel roles within site). In addition, since this is a secondary data analysis project, we were unable to simultaneously collect and analyze the data specific to our research. Despite these disadvantages, we were able to develop a model, or theory, grounded in the data associated with feedback acceptance and patient-management behavior. This reveals data collected for a specific purpose can provide the foundation for theory and model development in areas beyond the scope of the original research, and indeed strengthens the trustworthiness of the emergent model. Another limitation is that the clinical performance system physicians were asked to report on is specific to the VA. Although similar processes are used in non-VA medical centers to assess clinical performance, other performance measurement systems may enjoy greater credibility among their assesses and have a different impact on feedback recipients.

## Conclusions

Physicians’ acceptance of an existing VA performance assessment system is lukewarm, thereby impacting acceptance of feedback based on that system. Feedback acceptance is not only linked to the feedback, it is also related to the assessment process. There are specific aspects of A&F that induce and deter feedback acceptance and impact the incorporation of clinical practice changes that could result in enhancing performance and patient-management. Given this link between the two sides of the audit-and-feedback coin, developers of clinical performance measurement systems and feedback interventions should consider multiple aspects of audit and feedback, such as involving those being evaluated when establishing performance metrics and obtaining their views on feedback content and frequency. Without considering antecedents of feedback, lack of acceptance and ineffective feedback will likely be the consequent.

## Abbreviations

A&F, Audit and feedback; EPRP, External peer review program; PCP, Primary care provider; VAMC, Veterans Affairs Medical Center

## Figures and Tables

**Table 4:**
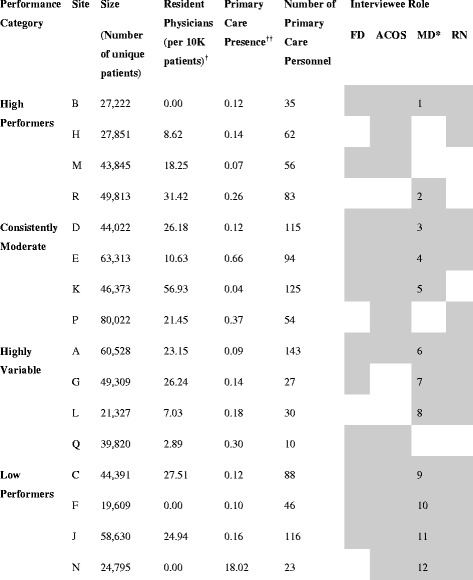
Primary Study Site Characteristics

Note: Grey boxes indicate we did conduct an interview for that role at that site.

Legend

FD – Facility Director

ACOS – Associate Chief of Staff

MD – Medical Doctor

RN – Register Nurse

* Participant interviews analyzed for this secondary study

† A measure of the strength of the facility’s academic orientation*.* Greater numbers mean a stronger academic orientation. 0 means no academic affiliation. *Source: Byrne et al., 2009* [37]

†† Operationalized as the percent of outpatient clinic stops provided at Community Based Outpatient Clinics. A low percentage indicates the majority of outpatient/primary care occurs at the main hospital, rather than at primary care clinic not located on the grounds of the main hospital. *Source: Byrne et al., 2009* [37]

## Appendix

### Appendix A

Table containing characteristics of sites included in the primary study including size of site (number of unique patients), number of Resident physicians (per 10,000 patients), primary care presence, number of primary care personnel and the role of the interviewee.

### Appendix B

Interview guide containing questions study participants answered.

**Table 5:** Primary Study Interview Guide

Number	Question
Q1	Tell me about your role at the VA.
*Q2 **	*In your efforts to provide the highest quality care that you can, how do you go about assessing the quality of care that you currently provide?*
Q3	How is clinical performance measured in your facility?
Q4	When you hear ‘EPRP’, what comes to mind?
Q5	How does EPRP fit in to the measurement of clinical performance at your facility?
Q6	Tell me about how you receive feedback about clinical performance at your facility. (If applicable, also, Tell me about how your PACT Team receives feedback about clinical performance.)
Q7	Tell me about the last time you received feedback about clinical performance. (If applicable, tell me about the last time your PACT team received feedback regarding its clinical performance.)
*Q8 **	*What do you personally do with the feedback when you get it?*
Q9	What are the consequences of feedback at your facility? (i.e., what happens if you are given feedback about your performance and you choose to ignore it)?
*Q10 **	*What could your facility be doing that they’re not doing now to help you track your individual clinical performance?*
Q11	To what extent has PACT been implemented at your facility?
Q12	Since the introduction of PACT at your facility, how has the measurement and assessment of clinical performance changed, if at all?
Q13	Since the transition to PACT, what changes have you noticed about the clinical performance information made available to you and your team? What has stayed the same?
Q14	How does your PACT teamlet use clinical performance information?
Q15	What could your facility be doing to better inform you about your PACT team's clinical performance?
Q16	Is there anything else that we have not discussed that you would like to share?

** Question analyzed for this study*
